# Eating Disorder Diagnoses in Children and Adolescents in Norway Before vs During the COVID-19 Pandemic

**DOI:** 10.1001/jamanetworkopen.2022.22079

**Published:** 2022-07-13

**Authors:** Pål Surén, Anne Benedicte Skirbekk, Leila Torgersen, Lasse Bang, Anna Godøy, Rannveig Kaldager Hart

**Affiliations:** 1 Norwegian Institute of Public Health, Oslo, Norway; 2 Nic Waals Institute, Lovisenberg Hospital, Oslo, Norway; 3 Department of Health Management and Health Economics, University of Oslo, Oslo, Norway

## Abstract

This cohort study assesses trends in diagnoses of eating disorders among girls aged 6 to 16 years in Norway before and during the COVID-19 pandemic.

## Introduction

Studies from the US^1,2^ and Canada^3,4^ have reported increasing numbers of children and adolescents receiving treatment for eating disorders during the COVID-19 pandemic. Most patients are girls,^1,2,4^ and the predominant diagnosis is anorexia nervosa.^1,2^ There is insufficient information about the magnitude of this increase on a population level. In this cohort study, we analyzed trends in diagnoses of eating disorders among children and adolescents in Norway before vs during the pandemic.

## Methods

We obtained primary care data from the Norwegian Registry for Primary Health Care and specialist care data from the Norwegian Patient Registry.^5^ Reporting to these registries is mandated by law and linked to the national reimbursement systems for health services. The entire Norwegian population is covered. This study followed the STROBE reporting guideline and was approved by the Norwegian Regional Committees for Medical and Health Research Ethics. Because the study used existing registry data, informed consent was not required.

Individuals aged 6 to 16 years living in Norway on January 1, 2020 (pandemic cohort), were observed from January 2019 to December 2021. The comparison group included this age group living in Norway on January 1, 2018 (prepandemic cohort), observed from January 2017 to December 2019. We assessed changes in the percentage of individuals with recorded eating disorders since onset of the pandemic in March 2020 in the pandemic cohort and after March 2018 in the prepandemic cohort. Changes were compared by difference-in-difference models.^6^ We calculated monthly percentages of individuals with eating disorders using event study models to estimate relative changes. Analyses were done by sex and age group (6-12 and 13-16 years) using Stata, version 16.0. The eMethods in the Supplement gives additional information.

## Results

The number of boys with eating disorder diagnoses was low; thus, girls were analyzed. The pandemic cohort included 348 187 girls (mean [SD] age, 11.03 [3.13] years), and the prepandemic cohort, 353 848 girls (mean [SD] age, 10.96 [3.15] years) (Table). For girls aged 6 to 12 years, we observed larger relative increases in the percentage with eating disorder diagnoses in the pandemic cohort: 66.90% (95% CI, 33.12%-100.67%) in primary care and 278.30% (95% CI, 160.44%-396.16%) in specialist care. For girls aged 13 to 16 years, the relative increase was 126.54% (106.48%-146.59%) in primary care and 95.96% (95% CI, 79.54%-112.38%) in specialist care. Increases were attributable to new cases.

**Table.** Recorded Eating Disorder Diagnoses Among Girls Aged 6 to 16 Years in the Pandemic and Prepandemic Cohorts^a^

Service type, age group	Pandemic cohort			Prepandemic cohort			Simple difference-in-difference estimate, % (95% CI)^b^
	Total No.	Girls with eating disorder diagnosis, No. (%)		Total No.	Girls with eating disorder diagnosis, No. (%)		
		January 2019 to February 2020	March 2020 to December 2021		January 2017 to February 2018	March 2018 to December 2019	
**Primary care**							
6-12 y	222 093	185 (0.08)	501 (0.23)	227 588	149 (0.07)	346 (0.15)	66.90 (33.12-100.67)
13-16 y	126 094	507 (0.40)	1554 (1.23)	126 260	478 (0.38)	884 (0.70)	126.54 (106.48-146.59)
**Specialist care**							
6-12 y	222 093	35 (0.02)	270 (0.12)	227 588	39 (0.02)	180 (0.08)	278.30 (160.44-396.16)
13-16 y	126 094	621 (0.49)	1907 (1.51)	126 260	499 (0.40)	1190 (0.94)	95.96 (79.54-112.38)

^a^ The pandemic cohort included girls living in Norway on January 1, 2020, and the prepandemic cohort included girls living in Norway on January 1, 2018. In primary care, eating disorders were defined by codes P11 (eating problem in child) and P86 (anorexia nervosa/bulimia) in the *International Classification of Primary Care, 2nd edition*. In specialist care, eating disorders were defined by code F50 (eating disorders) in the *International Statistical Classification of Diseases and Related Health Problems, Tenth Revision*.

^b^ The estimate equals the difference between the percentage change in the pandemic cohort and the percentage change in the prepandemic cohort, calculated as follows: ([(pandemic cohort postperiod percentage − pandemic cohort preperiod percentage) − (prepandemic cohort postperiod percentage − prepandemic cohort preperiod percentage)] / pandemic cohort preperiod percentage) × 100. The calculation of 95% CIs is described in the eMethods in the Supplement.

The monthly percentage of girls with eating disorder diagnoses increased over time in the prepandemic cohort (Figure). The pattern was similar in the pandemic cohort but with a disruption after onset of the pandemic. The monthly percentage of girls aged 13 to 16 years with an eating disorder diagnosis ranged from 0.05% to 0.08% before the pandemic and from 0.15% to 0.20% after onset in primary care and from 0.27% in February 2019 to 0.74% in December 2021 in specialist care. The relative increase ranged from −11.89% (95% CI, −47.23% to 23.46%) in March 2020 to 194.63% (95% CI, 160.27%-228.99%) in February 2021 for primary care and from 11.06% (95% CI, −2.84% to 24.96%) in May 2020 to 150.70% (95% CI, 128.87%-172.53%) in November 2021 for specialist care and was statistically significant throughout 2021.

**Figure.**
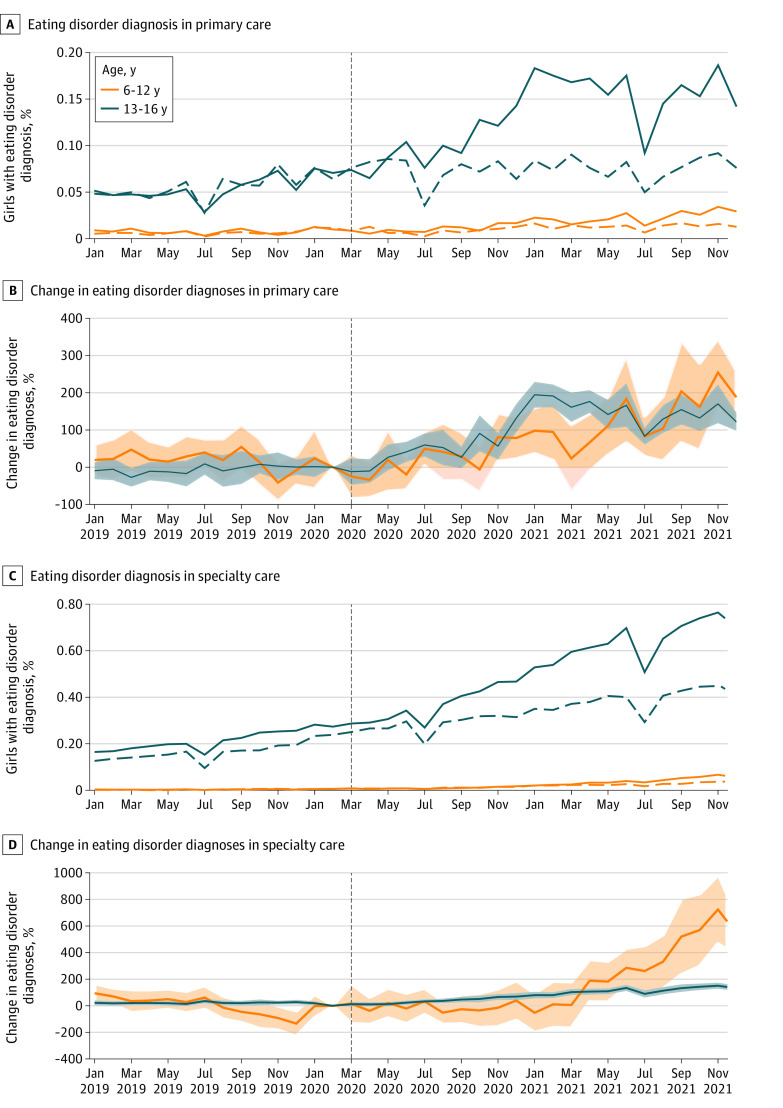
Girls With Recorded Eating Disorder Diagnoses in Norway Before vs During the COVID-19 Pandemic A-D, The unit of analysis was person-months. Vertical dashed lines indicate the onset of the COVID-19 pandemic in March 2020. A and C, Solid lines represent girls living in Norway on January 1, 2020 (pandemic cohort), with a recorded diagnosis of an eating disorder during the pandemic (January 2019 to November 2021), and dashed lines represent girls living in Norway on January 1, 2018 (prepandemic cohort), with a recorded diagnosis of an eating disorder before the pandemic (January 2017 to November 2019). Summer vacation may account for decreases in July. B and D, Changes in the percentage of eating disorder diagnoses in the pandemic cohort vs the prepandemic cohort (comparison group) are shown. Shaded areas indicate 95% CIs.

Among girls aged 6 to 12 years, the percentage with eating disorder diagnoses was lower but increased after March 2021. In specialist care, the percentage did not exceed 0.10%, but the relative increase remained greater than 200% after May 2021.

## Discussion

We found a substantial increase in the number of girls diagnosed with eating disorders in Norway starting after onset of the COVID-19 pandemic. The timing of the trend disruption suggests that the increase was associated with societal changes induced by the pandemic, including restrictions placed on youth’s lives, education, and activities. Limitations were that follow-up was incomplete for teenagers older than 16 years, we could not distinguish between eating disorder subtypes, and diagnostic data were not validated. Our findings are similar to those from North America,^1,2,3,4^ suggesting that the increase in eating disorders occurred internationally.

## Notes

Supplement.
eMethods
