# Supplementary material for: Eating Disorder Diagnoses in Children and Adolescents in Norway Before vs During the COVID-19 Pandemic
Source: JAMA Netw Open. 2022 Jul 13;5(7):e2222079. doi: 10.1001/jamanetworkopen.2022.22079 (PMC9280394; doi:10.1001/jamanetworkopen.2022.22079)
Supplement: Supplement. — eMethods [file jamanetwopen-e2222079-s001.pdf]

Supplemental Online Content

Surén P, Skirbekk AB, Torgersen L, Bang L, Godøy A, Hart RK. Eating disorder diagnoses in children and adolescents in Norway before vs during the COVID-19 pandemic. *JAMA Netw Open.* 2022;5(7):e2222079.
doi:10.1001/jamanetworkopen.2022.22079

eMethods.

This supplemental material has been provided by the authors to give readers additional information about their work.

© 2022 Surén P et al. *JAMA Network Open.*

eMethods

Data, study population and variables
The data set was derived from the emergency preparedness registry established by the Norwegian Institute of Public Health during the COVID-19 pandemic. Primary care data were obtained from the Norwegian Registry for Primary Health Care, and specialist care data were obtained from the Norwegian Patient Registry. Reporting to these registries is mandatory for health services receiving government reimbursements. We had access to consultation data for the years 2017-2021.

The registries are likely to capture nearly all diagnosed cases of eating disorders in Norway. In primary care, eating disorder diagnoses are recorded by general practitioners. In specialist care, eating disorders among individuals under 18 years are recorded by hospitals and outpatient child and adolescent mental health services.

Our study population was defined by the following inclusion criteria, that had to be met in 2020 for the pandemic cohort and 2018 for the pre-pandemic cohort:

- Residency in Norway on January 1st.
- Being in the age range of 6-16 years on December 31st.
- Having a valid and permanent personal identification number, to allow for linkages between registries.

The lower age restriction corresponds to mandatory school starting age in Norway. The upper age restriction is linked to limitations of our data set. At age 18, specialist care for mental health is transferred to adult clinics. Our specialist care data for adults were not complete for 2021. To avoid losing study subjects to follow-up because they transfer to adult services, we only included individuals who were younger than 18 years throughout the observation period. For consistency, we applied similar age restrictions to the primary care data.

Our findings are reported according to the STROBE guidelines for cohort studies.

The study has been approved by the Norwegian Regional Committees for Medical and Health Research Ethics (REC), approval number 2021/267200. We confirm that all administrative permissions have been granted to access and use the data for this study. As the study utilized existing registry data, no written or verbal consent to participate was required.

Sex was reported according to the information provided by the Norwegian Population Registry. For most individuals, this is the sex registered at birth. For some individuals, registered sex may have changed after birth, either because the individual has requested such a change or because the registration was erroneous in the first place. The data set did not contain information about whether registered sex had ever been changed.

Estimation of difference-in-difference models (results in Table)
To formally compare the trend development in the pandemic and pre-pandemic cohort, we used two methodological strategies. First, we calculated a simple difference-in-difference (DiD) estimate. Letting $\bar{X}$ denote the mean consultations, the simple DiD estimate is defined as (Angrist & Pischke, 2014):

$$DiD = (\bar{X}_{Pandemic,post} - \bar{X}_{Pandemic,pre}) - (\bar{X}_{Pre-pandemic,post} - \bar{X}_{Pre-pandemic,pre})$$

This estimate captures the change in the percentage of individuals with at least one consultation in the pandemic cohort, minus the same change in pre-pandemic cohort, and is shown in the Table.

© 2022 Surén P et al. *JAMA Network Open.*

To obtain confidence intervals for the DiD estimate, we conducted a simple DiD regression analysis. The regression model was fitted on a panel data set, including one pre- and one post observation for each individual in the pre-pandemic and pandemic cohort. The simple DiD estimate is equivalent to β_{DiD} estimated by the following regression equation (Angrist & Pischke, 2014), where $y_{i,t}$ takes 1 if the individual had a consultation in the given period, otherwise 0:

$$y_{i,t} = \beta_{Pandemic} X_{Pandemic\ cohort,i} + \beta_{Post} X_{Post,i,t} {}^{*} \beta_{DiD} (X_{Pandemic\ cohort,i} + X_{Post,i,t}) + \ \varepsilon_{i,t}$$

Standard errors are robust, and clustered at the level of the individual. 95% confidence intervals are then calculated using the standard formula: $CI = \bar{X}\ \ += \ 1.96 * SE_{\beta_{DiD}}$

We also stratified the sample according to whether an individual was a new or recurrent case. Individuals in the pandemic cohort were defined as recurrent cases if they had any eating disorder consultations in 2019, otherwise as new cases. The same classification was made for the pre-pandemic cohort using data from 2017. The assessment of new versus recurrent cases were made separately for primary and specialist care. Note that in the stratified models, the pre-period was then shortened to two months (January and February in 2020 for the pandemic cohort, and the same months in 2018 for the pre-pandemic cohort).

Estimation of event study models (results in Figure)
In a data set with person-months at the unit of analysis, we fitted event study models, with results shown in the Figure (Borusyak, Jaravel & Spiess, 2021). The monthly propensity of treatment was the outcome. The event study estimates capture both changes in number of individuals in treatment, and changes in the duration of treatment for those already in treatment. We estimated the coefficients of the following regression equation using Ordinary Least Squares (OLS) regression:

$$y_{i,t} = \sum_{k=-14,k!=-1}^{21} X_{Intervention} * 1(t - t0 = k)\beta_k \ \ + \sum_{Y=-1}^{1} \beta_{Year} \mathrm{X}_{Year,i,t} \ \ + \sum_{W=1}^{12} \beta_{Month} \mathrm{X}_{Month,i,t} \ \ + \boldsymbol{\beta}\mathbf{X}_{\ \boldsymbol{i,t}} + \ \varepsilon_{i,t}$$

Here, t0 refers to the first month of lockdown, k counts months forward and backwards from the reference point. For each of the k months, the expression X_Intervention* 1(t−t0=k) constructs a variable taking on 1 if the observation was in the pandemic cohort, k months away from March 2020, otherwise 0. K-1 is the omitted reference category, and this value was assigned to all observations in the pre-pandemic cohort.

A set of dummy variables for month net out seasonal variation in consultations. To handle increases over period within each cohort, we control for duration since time zero in years, e.g., -1 will refer to 2019 in the pandemic cohort and 2017 in the pre-pandemic cohort. The vector of controls X includes dummies for region and a variable running from 0 to 1 showing the proportion of Easter vacation falling into the given month in the given year. (Consultation numbers during those days are very low.)

The coefficients of interest, plotted in the Figure, are the βk's. These give month-by-month estimates of how the development in the pandemic cohort deviated from the development in the pre-pandemic cohort. βk's for the period before lockdown (March 2020 in the pandemic cohort, March 2018 in the pre-pandemic cohort) indicate whether trends in the pandemic and pre-pandemic cohort were parallel prior to the pandemic. Parallel trends prior to the pandemic combined with deviations in trends after the onset of the pandemic suggest that societal changes related to the pandemic had an impact on the outcome.

© 2022 Surén P et al. *JAMA Network Open.*

Literature

Angrist, J. D., & Pischke, J. S. (2014). *Mastering'metrics: The path from cause to effect*. Princeton university press.

Borusyak, K., Jaravel, X., & Spiess, J. (2021). Revisiting event study designs: Robust and efficient estimation. *arXiv preprint arXiv:2108.12419*.

© 2022 Surén P et al. *JAMA Network Open.*
